# Factors influencing treatment duration of oral propranolol in infantile hemangioma: a five-year retrospective analysis

**DOI:** 10.3389/fped.2025.1693660

**Published:** 2026-01-22

**Authors:** Qian Li, Liu Xiao, Mengting Su, Hongguang Chen, Haihua Zhang, Nana Cao, Lixiao Jiang, Xiaoyan Liu, Gaolei Zhang

**Affiliations:** 1 Department of Dermatology and Venereology, Capital Center for Children’s Health, Capital Medical University, Beijing, China; 2 Key Laboratory of Mental Health, Ministry of Health (Peking University), National Clinical Research Center for Mental Disorders (Peking University Sixth Hospital), Peking University Sixth Hospital, Peking University Institute of Mental Health, Beijing, China

**Keywords:** infantile hemangioma, propranolol, efficacy, influence factor, hemangioma

## Abstract

**Background:**

Oral propranolol is effective in promoting the involution of infantile hemangiomas (IHs), but treatment outcomes vary widely.

**Objectives:**

To identify demographic and clinical factors influencing the time to achieve complete regression in IH patients treated with propranolol.

**Methods:**

A retrospective study was conducted on 410 IH patients treated at the Department of Dermatology and Venereology, Capital Center for Children's Health, Capital Medical University, between 2018 and 2023. Patients received propranolol (3 mg/kg/day) and were followed monthly. The primary outcome was the time (months) required to achieve an Achauer grade IV response, defined as complete or near-complete resolution. Treatment was continued until this endpoint was reached.

**Results:**

The cohort included 157 males (38.3%) and 253 females (61.7%) with a median age of 2 months (interquartile range, 2–4 months); 36 (8.8%) were preterm. All patients eventually achieved an Achauer IV outcome, with a median treatment duration of 7 months (95% CI, 6–10 months). Age at treatment initiation, lesion location, and IH subtype significantly influenced the time to cure, while sex and prematurity did not.

**Conclusions:**

Propranolol remains the first-line pharmacotherapy for IHs. The treatment duration required to reach an Achauer IV outcome is prolonged when therapy begins after 7 months of age, when lesions are located in the periorbita, nose, chest/back, perineum/anal region, or when deep/mixed subtypes are present.

## 1 Introduction

Infantile hemangiomas (IHs) represent a category of benign vascular neoplasms. IHs are benign vascular tumours that affect up to 10% of infants and arise in the first few weeks-to-months of life (1). IHs can be classified into several types based on their distribution patterns, namely single, multiple, segmental, and intermediate types. Moreover, according to their morphological features and clinical depth, they can be further categorized as superficial, deep, and mixed types (2). IHs exhibit a well—defined developmental trajectory and undergo three distinct stages: proliferation, plateau, and involution. The most rapid growth phase of IHs generally takes place within the initial 8 weeks after birth, and they usually attain their maximum size at around 9–12 months of age. After a relatively brief plateau period, IHs start to gradually regress after the first year of life. Nevertheless, the speed of involution shows considerable variation from one individual to another (3). In approximately 69% of untreated cases, residual lesions can be observed in hemangiomas (4). Although a considerable number of IHs are small and pose no significant threat, a notable proportion of them necessitate active medical intervention. During the rapid proliferation stage, IHs may give rise to severe complications, such as pain, ulceration, functional limitations, and cosmetic disfigurement (2), some are associated with complications that can be life-altering or life-threatening, and permanent sequelae can be psychologically distressing (5). The optimal timing, duration, and discontinuation administration for treatment remain controversial; additionally, uncertainties persist regarding the long-term prognosis of IH patients. Furthermore, the influence of demographic factors (including gender, gestational age, and birth weight), lesion subtypes (focal, segmental, or multiple), and anatomical location on the risk of complications, recurrence rates, and aesthetic outcomes is not yet fully elucidated. This lack of comprehensive understanding often results in clinical decision-making being primarily based on empirical judgment, even though most IHs resolve spontaneously, approximately 12%–15% of cases.

The previous study has demonstrated that several risk factors are associated with the development of complications in IHs. These factors include segmental distribution, deep tissue involvement, as well as the location on the face and neck (6, 7). Segmental and facial IHs show a high risk of unaesthetic sequelae, complications, and recurrence (7). Deep—seated IHs tend to make their appearance somewhat later in comparison to their superficial counterparts and have a marginally extended growth period. For sizable tumors that exhibit deep—tissue invasion, particularly those located in the parotid region, they may persist in growing until the patient reaches the age of 2. However, there is a spontaneous regression trend. Specifically, in 30% of patients, these tumors will partially or completely disappear by the time they are 3 years old. By the age of 5, this proportion drops to 20%, and by the age of 9, between 30% and 40% of patients will experience partial or complete regression of these tumors. Superficial and deep hemangiomas left significantly fewer complications than segmental and mixed hemangiomas (8). Conversely, deep and mixed hemangiomas left behind significantly more fibrofatty tissue than superficial hemangiomas, which cause functional impairment and cosmetic disfigurement (9). There is a window of opportunity to treat IHs before the rapid proliferative phase. The primary objectives of IH treatment are to inhibit the proliferation of vascular endothelial cells, promote tumor regression, and minimize tumor residue, ultimately improving the prognosis and quality of life for affected patients.

At present, the therapeutic effectiveness of propranolol, along with its underlying action mechanism, has been clarified to a relatively large extent. Nonetheless, multiple factors can impact how well propranolol works. These include the age when treatment is first started, the length of the treatment course, and the specific anatomical location of the hemangiomas. Furthermore, it is rather common for hemangiomas to experience rebound growth once the use of the drug is halted. Additionally, a small proportion of IHs exhibit resistance to propranolol (2). Research findings indicate that there exists a notable disparity in the probability of disease recurrence depending on when the treatment is discontinued. Specifically, there is a significant difference between stopping the therapy before the patient reaches 9 months of age and after they turn 24 months old. Apart from starting the treatment early, no other characteristics are linked to increased efficacy of propranolol (10).

A distinct and well—established link exists between starting treatment early and achieving better therapeutic outcomes. In particular, a significant contrast in treatment responses can be observed based on the age at which intervention commences. When the treatment was initiated in children aged between 18 and 27 months, 45% of them demonstrated suboptimal responses. In stark contrast, only 7% of children showed inadequate responses when the treatment was started within the first 0–2 months of life (11). For oral propranolol administration, when it is given to children beyond 6 months of age and up until they reach 12 months old, it can substantially elevate the regression rate of IHs. Moreover, the positive effects of this treatment can persist for as long as 3 months after the therapy has been discontinued. However, a rebound growth rate of 32% has been reported to occur in most patients within 2 months following propranolol withdrawal, yet retreatment has proven effective. Approximately 24% of patients were judged as requiring retreatment. Clinically, only 50% of patients started treatment before the age of 3 months, and this fact may have contributed to complications. Studies have demonstrated that children starting treatment before 4 months of age exhibit a better response to treatment, and those beginning treatment at an older age require prolonged treatment. It is certain that for IH cases at high risk of complications and recurrence, propranolol treatment should be prolonged. Nevertheless, the mechanisms underlying IH recurrence after propranolol treatment have not been completely elucidated (12).

While numerous research endeavors have elaborated on the therapeutic strategies for treating IHs, a contentious debate still lingers regarding the most opportune moment to initiate treatment, especially concerning the cessation of propranolol administration. At present, the medical community has yet to reach a unanimous agreement on the ideal duration for which propranolol should be prescribed to treat IHs. Furthermore, the existing body of literature offers limited accounts of long—term follow—up studies on IH patients who have undergone oral propranolol therapy. Our study aimed to conduct a comprehensive analysis encompassing various aspects such as the initiation and cessation of therapy, treatment duration, sex, anatomic location, and IH subtypes, as well as the clinical and therapeutic features of IH patients. Moreover, we sought to identify the conditions that are associated with complications, recurrence, and unsatisfactory sequelae. In order to prevent recurrence and reduce complications, future research is needed. These studies should focus on determining whether higher doses of propranolol or an extended administration phase would be beneficial. A better understanding of the natural course of IHs and related complications is crucial, which will not only help to enhance further research on them, but also contribute to the advancement of IH treatment strategies.

## 2 Data and methods

### 2.1 Patients

We conducted a retrospective review of patients with infantile hemangiomas (IHs) who received oral propranolol at the Department of Dermatology and Venereology, Capital Center for Children's Health, Capital Medical University, between 2018 and 2022. Patients were included if they met all of the following criteria: Diagnosis of IH according to the International Society for the Study of Vascular Anomalies (ISSVA) classification; Presence of a single hemangioma lesion; Initiation of oral propranolol therapy before 12 months of age; No prior treatment for IH; High adherence and complete follow-up, defined as continuous propranolol administration without interruptions exceeding 7 consecutive days and attendance at all scheduled clinical visits (at intervals of 4–6 weeks), with comprehensive documentation in medical records and photographs.

Exclusion criteria were as follows: Severe underlying conditions, including hepatic dysfunction, bronchial asthma, congenital bradycardia, or advanced atrioventricular block; Known hypersensitivity to propranolol; Guardians' refusal to participate.

### 2.2 Treatment and follow-up

All children included in the study started propranolol treatment with an initial dose of 1.0 mg/kg/d. For those who didn't exhibit any significant adverse reactions within a 7-day period, the dose was subsequently raised to 2.0 mg/kg/d, and finally up to 3 mg/kg/d. Regular follow-up appointments were performed monthly to evaluate both the therapeutic efficacy and potential adverse effects. During each follow-up, we took pictures to record the color and texture changes of IHs, and used B-ultrasound to measure the layer, size, blood flow, and boundary of the tumor. Treatment could be halted once the IHs had largely faded or the blood flow signal typically dropped below 20%. Prior to stopping the treatment, the dosage was progressively lowered. Initially, it was cut down to 2.0 mg/kg/d, and then, after a month, it was further reduced to 1.0 mg/kg/d. If no resurgence of IHs was seen after a month of taking the 1.0 mg/kg/d dose, patients were considered ready to end the treatment. The entire observation period was 36 months. If the tumor volume shrinks by more than 75% during this period, it is defined as effective treatment, and drug reduction could be initiated until discontinuation. Patients were followed up for an additional 6 months after the end of treatment to determine rebound.

### 2.3 Outcomes

The anatomical distribution of infantile hemangiomas (IHs) was categorized into eight predefined regions, each assigned a unique numerical code (“Location 1” to “Location 8”) to facilitate standardized data recording and statistical analysis. The corresponding anatomical sites were defined as follows:

Location 1—Scalp;
Location 2—Periorbital region;
Location 3—Nose;
Location 4—Face and neck;
Location 5—Extremities;
Location 6—Chest and back;
Location 7—Abdomen/loin and buttock;
Location 8—Perineum and anal region.

This coding system was consistently applied across patient enrollment records, follow-up documentation, and subsequent statistical analyses (including the Cox proportional hazards model).

Based on morphological characteristics, IHs were further classified into three subtypes: superficial, deep, and mixed. Superficial IHs typically exhibit bright red, raised, strawberry-like lesions; deep IHs involve subcutaneous tissue with minimal surface color change; and mixed IHs present both superficial and deep components.

Treatment efficacy was assessed using the Achauer grading system (7), which categorizes therapeutic outcomes as follows: Grade I (unsatisfactory, 0%–25% regression), Grade II (fair, 26%–50%), Grade III (satisfactory, 51%–75%), and Grade IV (excellent, >75% regression). A Grade IV response was defined as a complete or near-complete resolution and was considered an effective treatment outcome.

### 2.4 Statistical analyses

This study used survival analysis and the COX risk ratio regression model conduct the data processing. First, the overall cure rate curve and its associated feature distribution were plotted to estimate the median curative time. At each time point, the cure probability was determined using the Kaplan–Meier approach, and the log-rank test was employed to assess whether the differences between the two groups were statistically significant. Following this, the COX proportional hazards regression model was utilized to identify risk factors linked to the cure rate, compute the hazard ratio (HR), and establish the 95% confidence interval (CI). All data were initially recorded in Excel. Then, data collection and processing were performed using STATA Software. A *P* value below 0.05 on both sides was deemed to indicate statistical significance.

This research received approval from the Medical Ethics Committee at the Capital Center for Children's Health, affiliated with Capital Medical University. All diagnostic procedures and treatments were authorized by the patients or their family members, who also signed informed consent forms.

## 3 Results

### 3.1 General characteristics and disease characteristics

Among the 410 patients with infantile hemangioma participating in this study, 157 (38.3%) were boys and 253 (61.7%) were girls, yielding a male-to-female ratio of 1:1.6. Furthermore, 36 patients (8.8%) were born prematurely. Regarding the age at treatment initiation, 94 (22.9%) patients were 0 to 1 month old, 192 (46.8%) were aged 2 to 3 months, 93 (22.7%) were 4 to 6 months old, and 31 (7.6%) were older than 7 months. Their median age at the treatment initiation were 2 months (interquartile range, 2 to 4 months) (Table 1).

**Table 1:** Characteristics of the patients.

Characteristic	Group	*N* = 410 (%)
Sex	Male	157 (38.3)
	Female	253 (61.7)
Is it premature		
	Yes	36 (8.8)
	No	374 (91.2)
Age at treatment initiation(month)		
	0–1	94 (22.9)
	2–3	192 (46.8)
	4–6	93 (22.7)
	≧7	31 (7.6)
Location		
	scalp	73 (17.8)
	periorbita	33 (8.0)
	nose	22 (5.4)
	face and neck	71 (17.3)
	extremities	100 (24.4)
	chest and back	38 (9.3)
	abdomen/loin and buttock	52 (12.7)
	perineum and anal region	21 (5.1)
Subtype	superficial	263 (64.1)
	deep	51 (12.4)
	mixed	96 (23.4)

**Figure 1:**
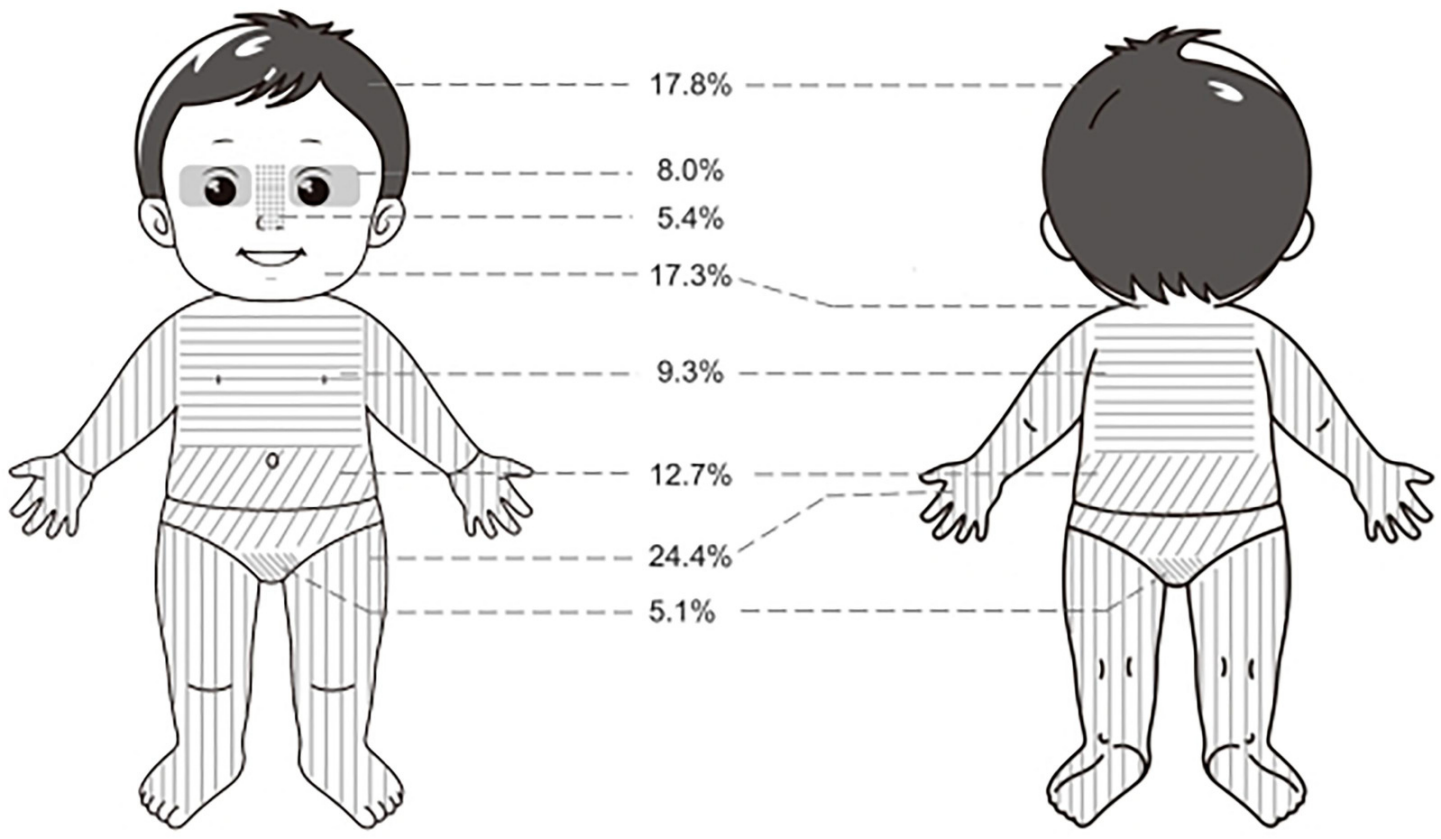
Locations of IHs.

As illustrated in Figure 1, the anatomical distribution of hemangiomas was summarized as follows for descriptive purposes: the head and face were the most commonly affected areas, accounting for 48.5% of cases, including the scalp, periorbita, nose, face, and neck. The extremities were affected in 24.4% of cases, and the trunk in 12.7%. It should be noted that while these regions are grouped for summary, the Cox proportional hazards model analyzed each specific location (scalp, periorbita, nose, face, neck, extremities, trunk) separately to assess their individual effects on treatment duration.

Based on ISSVA morphology classifications, 263 patients (64.1%) presented with superficial hemangiomas, 51 patients (12.4%) had deep hemangiomas, and 96 patients (23.4%) exhibited mixed hemangiomas.

Treatment was continued until each patient achieved an Achauer grade IV outcome, which was defined as complete or near-complete resolution of the lesion. The primary endpoint was the time (in months) required to reach this grade.

**Figure 2:**
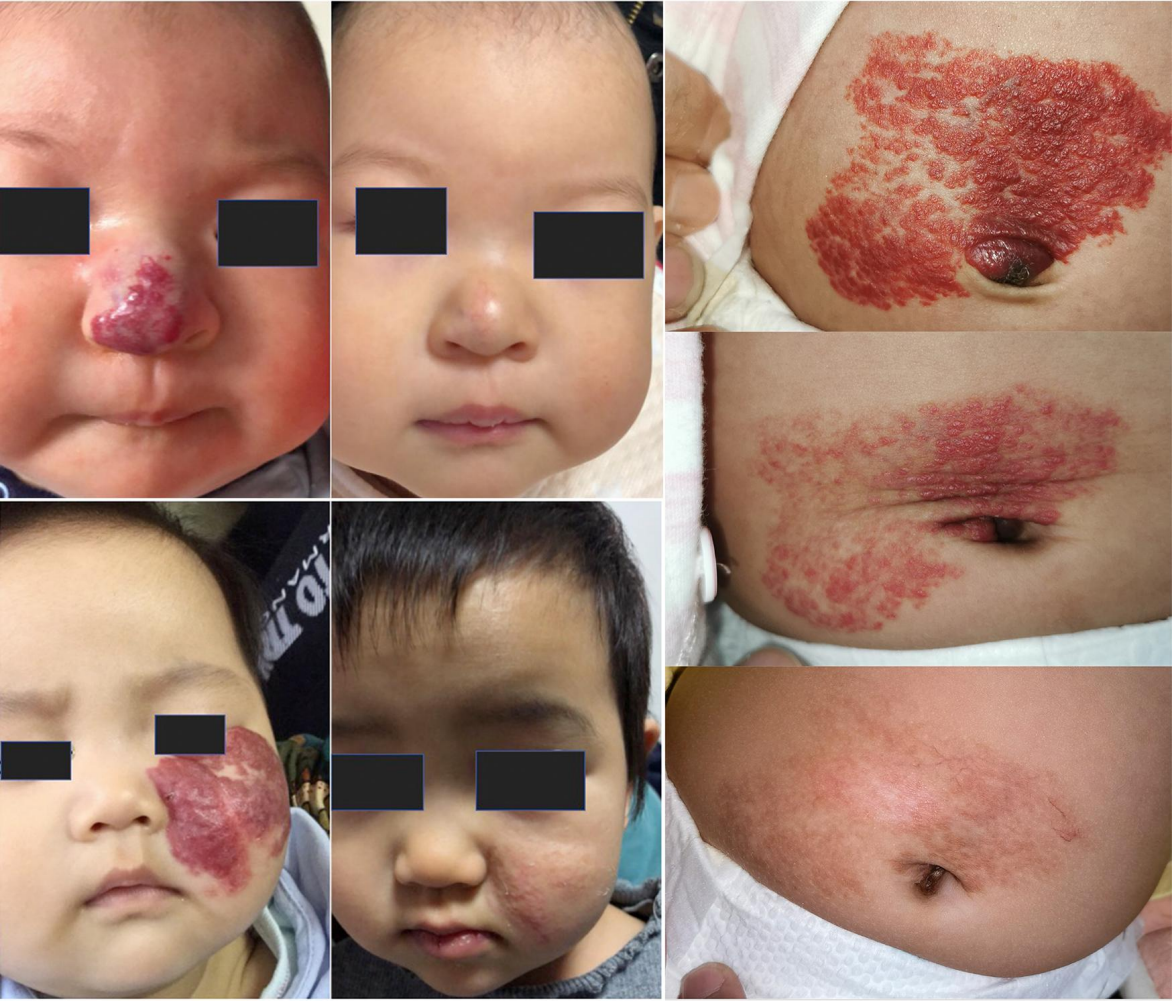
Treatment effect.

All 410 children eventually achieved Achauer IV, with a median time to endpoint of 7 months (95% CI: 6–10 months) (Figure 2, Table 1).

During the 6-month follow-up period after propranolol discontinuation, 17 children (4.1%) experienced varying degrees of recurrence. Among them, 10 cases were deep type, 5 were mixed, and 2 were superficial. Recurrences occurred at the periorbita (3 cases), nose (2), face (2), extremities (3), chest/back (3), and perineum/anal region (4).

### 3.2 Cure rate curve estimation and distribution of treatment duration

**Figure 3:**
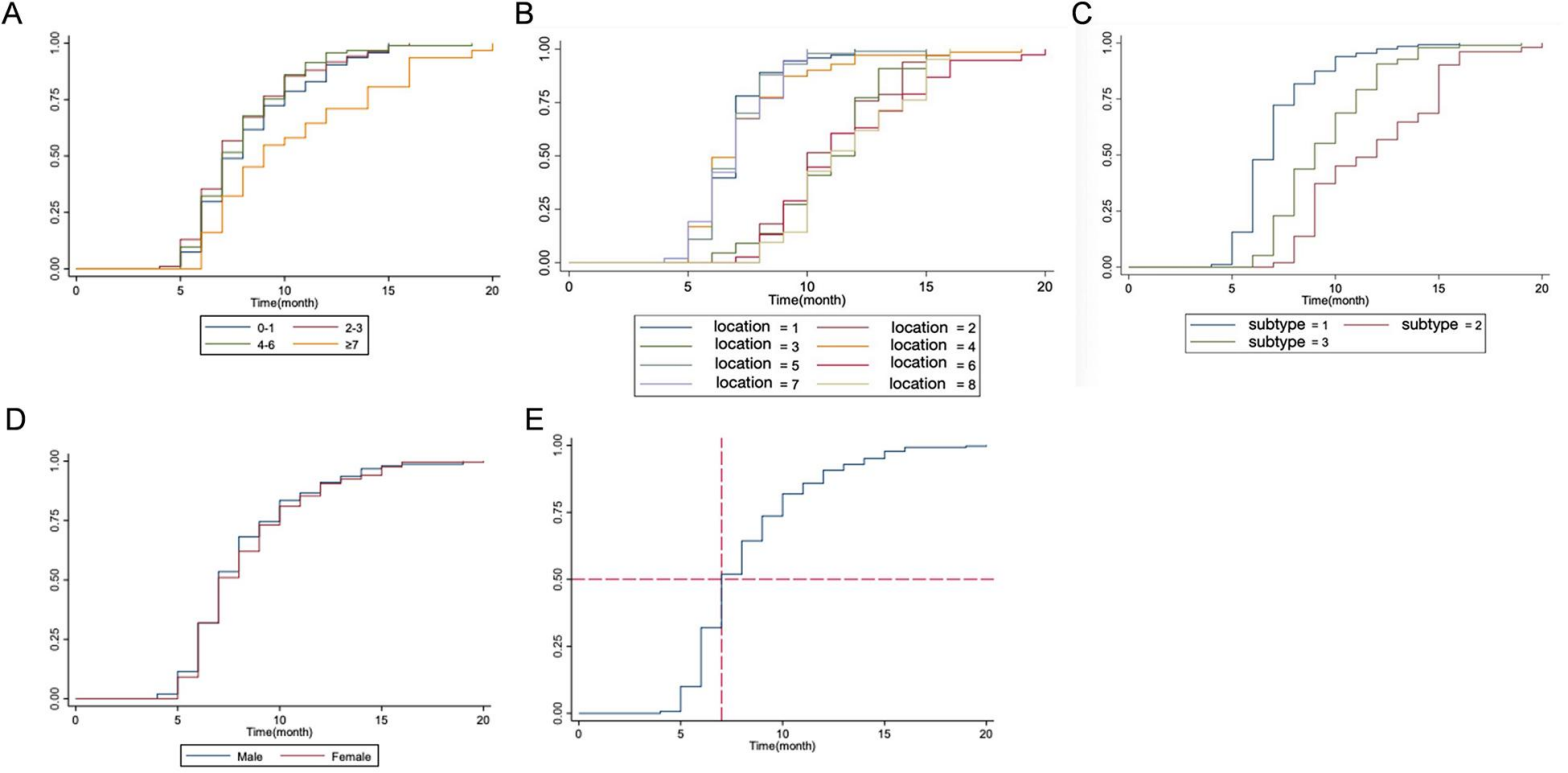
Cure rate curves and prognostic factors in infantile hemangiomas (IHs) treatment. Panel **(A)** Cure rate stratified by age at treatment initiation. Log-rank test, *P* < 0.001, indicating age significantly affects cure rate. Panel **(B)** Cure rate by lesion location. Log-rank test, *P* < 0.001, showing location is a key prognostic factor. Panel **(C)** Cure rate by IHs subtype. Log-rank test, *P* < 0.001, demonstrating subtype influences treatment outcomes. Panel **(D)** Cure rate comparison by gender (male vs. female). Log-rank test, *P* = 0.455, suggesting no gender-related difference in cure rate. Panel **(E)** Cure rate stratified by prematurity status. Log-rank test, *P* = 0.670, indicating prematurity does not significantly affect cure rate.

The median treatment duration for all subjects was 7 months [95% confidence interval(CI): 6–10 months]. The analysis of cure rate curve showed statistically significant differences existed with respect to the age at treatment initiation (*P* < 0.001), location (*P* < 0.001) and subtypes of IHs (*P* < 0.001). Conversely, there were no notable differences found in terms of gender (*P* = 0.455) or prematurity (*P* = 0.670) (Figure 3).

### 3.3 Cox proportional hazards model analysis of prognostic factors for children with hemangiomas

In this study, a Cox proportional hazards model was used to identify prognostic factors associated with time to cure in children with infantile hemangiomas, using the first category as the reference group. The results showed that for each additional month of age at treatment initiation, the hazard of achieving cure decreased by approximately 7% (HR = 0.93 per month increase in age, 95% CI: 0.90–0.96, *p* < 0.001), indicating that older patients required a longer duration of propranolol therapy to achieve complete resolution.

Regarding anatomical locations, hemangiomas located at sites 2, 3, 6, and 8 exhibited significantly lower cure rates compared with those on the scalp (HR = 0.18, 0.28, 0.17, and 0.19, respectively; all *p* < 0.001), indicating a longer time to cure. No significant differences were observed for locations 4, 5, and 7.

**Figure 4:**
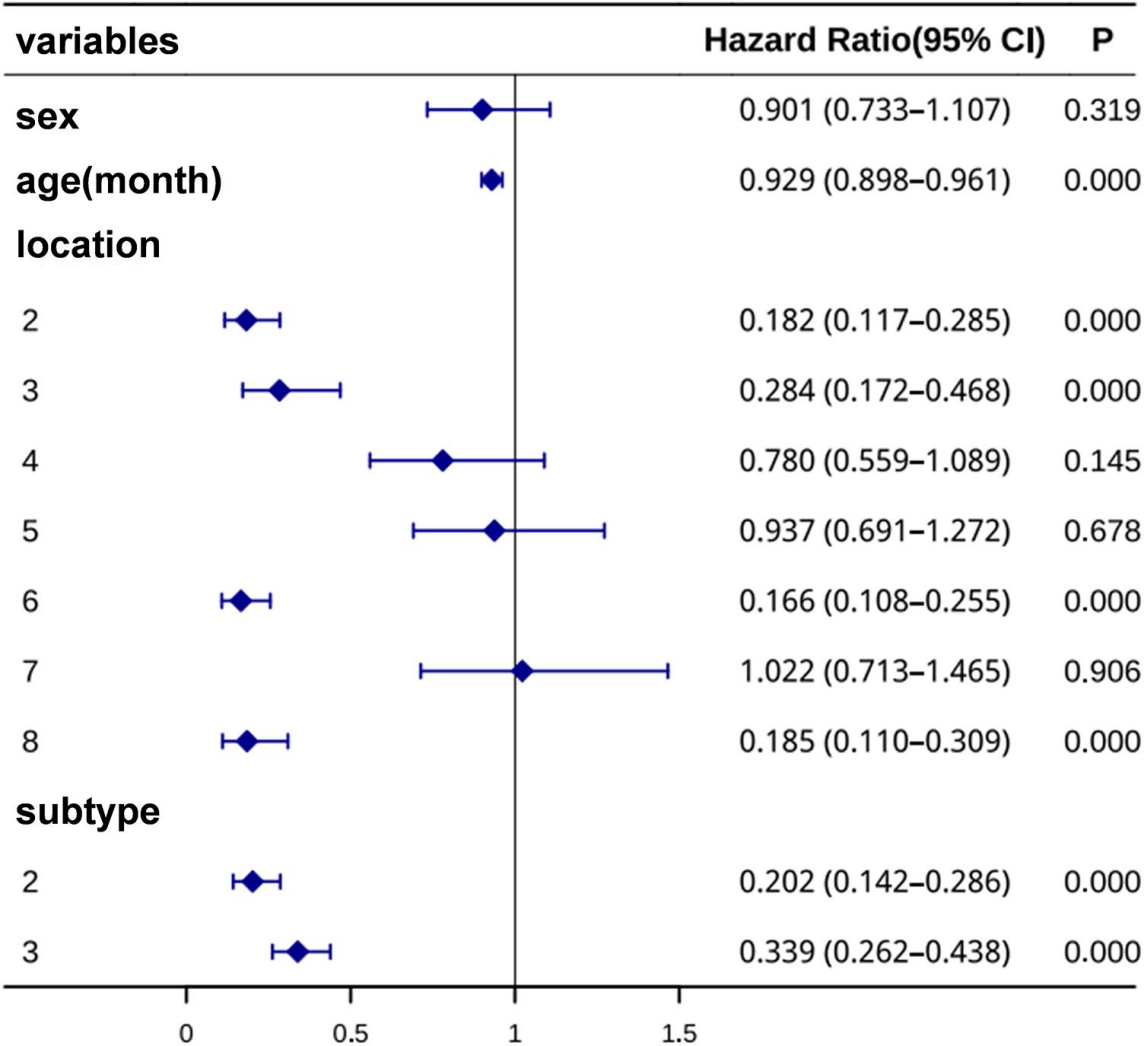
Cox proportional hazards model analysis of prognostic factors for children with hemangiomas.

In terms of morphological subtype, both deep (HR = 0.20, *p* < 0.001) and mixed (HR = 0.34, *p* < 0.001) hemangiomas showed lower cure rates compared with superficial types, reflecting slower regression. Gender was not a significant predictor (HR = 0.90, *p* = 0.319) (Figure 4).

## 4 Discussion

IH is a benign vascular tumor arising from excessive endothelial proliferation during embryonic development (7). Its pathogenesis remains incompletely understood, involving both intrinsic and extrinsic mechanisms. Intrinsic hypotheses suggest that IH originates from uncontrolled proliferation of progenitor cells, either due to local genetic mutations or recruitment from distant sources such as the placenta or bone marrow. Extrinsic theories emphasize environmental triggers, including hypoxia, the renin–angiotensin system (RAS), and cytokine signaling. These mechanisms likely act in concert, reflecting a multifactorial process in IH formation (10).

The predominant model proposes that immature endothelial cells migrate to the lesion site and, under stimulation by angiogenic factors, differentiate into endothelial cells, pericytes, and adipocytes (11). Among these factors, vascular endothelial growth factor (VEGF) plays a central role in promoting endothelial proliferation, angiogenesis, and survival (12, 13). Matrix metalloproteinases (MMPs), particularly MMP2 and MMP9, facilitate extracellular matrix degradation and vascular remodeling (14). Their activity is tightly regulated by tissue inhibitors of metalloproteinases (TIMPs), especially TIMP-1, maintaining extracellular matrix homeostasis.

Clinically and histologically, IH undergoes a characteristic life cycle comprising proliferating, involuting, and involuted phases (15). It usually appears shortly after birth and enters a rapid growth phase during infancy, marked histologically by densely packed, immature endothelial and pericytic cells forming disorganized vascular channels. The involuting phase begins around one year of age, during which endothelial cells mature, apoptosis increases, and the lesion gradually regresses with reduced vascularity and color. In the final involuted phase, most vascular structures are replaced by adipose and fibrous tissue (10). The expression of angiogenic factors such as VEGF, bFGF, MMP2, and MMP9 is elevated during proliferation and declines during involution, while anti-angiogenic regulators like TIMPs show the opposite trend (16, 17). The regression process may involve the differentiation of mesenchymal stem cells into adipocytes and fibroblasts, as well as immune-mediated clearance of proliferating endothelial cells. Collectively, these findings support a dynamic balance between angiogenic activation and immune-driven regression throughout IH progression (18).

Traditionally, IH treatment relied on physical modalities such as laser therapy, cryotherapy, and systemic corticosteroids. In severe or refractory cases, agents like vincristine, α-interferon, or cyclophosphamide were used but carried significant adverse effects. Recently, oral propranolol has emerged as a highly effective and well-tolerated first-line therapy for complicated IHs (15). Propranolol, a non-selective β-adrenergic blocker, exerts therapeutic effects through multiple mechanisms (19–22): (I) inducing pericyte-mediated vasoconstriction via β2-adrenergic receptor inhibition; (II) suppressing angiogenesis by downregulating VEGF, bFGF, MMP2/9, and IL-6; (III) inhibiting endothelial cell proliferation and promoting apoptosis; and (IV) facilitating the differentiation of progenitor cells toward adipocytes, thereby accelerating lesion regression (15). Through these combined actions, propranolol effectively shifts IHs from the proliferative to the involuting phase.

In this study, infantile hemangiomas (IHs) occurred predominantly in females, with a female-to-male ratio of 1.6:1, and were more frequent in premature infants (8.8%) than in the general population. Earlier studies have shown that the incidence of IH increases as gestational age decreases (19), and that female sex and preterm birth are major risk factors for IH (23). The higher incidence among females is thought to be related to estrogen-mediated angiogenesis. Estrogen can promote angiogenesis through several mechanisms (24–27): (I) enhancing the expression of hypoxia-inducible factors (HIFs) and estrogen receptors in vascular endothelial cells, thereby stimulating angiogenic factor production; (II) increasing mitotic activity and DNA synthesis in endothelial cells; (III) preventing endothelial apoptosis and promoting the expression of adhesion molecules and integrins; (IV) stimulating the synthesis of growth factors such as VEGF, bFGF, IGF, and TGF-β; and (V) modulating macrophage secretion of proangiogenic factors that facilitate hemangioma progression.

Preterm birth is another important risk factor, largely attributable to hypoxia-related mechanisms during fetal development. Premature infants are more likely to experience hypoxia, which activates HIF-1α and subsequently upregulates downstream targets such as VEGF, IGF-2, and GLUT-1, promoting vascular proliferation in IH. Kleinman et al. reported increased HIF-1α expression in IH tissues and proposed that it drives the production of VEGF, MMP9, and SDF-1α, thereby facilitating the recruitment of circulating endothelial progenitor cells during postnatal angiogenesis (28). Furthermore, hypoxia may cause placental-derived endothelial progenitor cells to enter the fetal circulation and colonize specific sites in the body, though the precise mechanisms remain unclear and warrant further investigation (29).

Clinically, superficial IHs located on the head and face were the most frequent manifestations, consistent with previous reports (25). Oral propranolol remains the first-line treatment, with a median time to achieve complete regression of approximately 6 months. Our findings emphasize the importance of early initiation of propranolol—preferably before 7 months of age—as delayed treatment initiation was associated with longer treatment duration. This may reflect the reduced proliferative potential of hemangioma cells as lesions mature, as well as differences in cellular origin or microenvironment across IH subtypes and anatomical sites. Recent evidence suggests that a complex interplay exists between mutated progenitor cells (of uncertain origin) and their surrounding microenvironment, which may partly explain these variable therapeutic responses (27).

Remarkably, all IHs in this study achieved Achauer grade IV resolution, with a recurrence rate of only 4.1%, substantially lower than the 10%–17% reported in previous studies (30). The lower recurrence rate in our cohort may be attributed to earlier treatment initiation, standardized dosing, and close follow-up, underscoring the clinical value of early and well-monitored intervention.

No adverse reactions were observed during propranolol therapy in this study, consistent with recent large-scale studies showing that propranolol is generally safe and well tolerated, with only mild, reversible side effects such as transient bradycardia or sleep disturbance (30). The absence of complications in our patients may be related to strict dose escalation monitoring and regular follow-up, highlighting the safety of propranolol under standardized treatment protocols.

In summary, our results confirm that female sex and preterm birth are key risk factors influencing IH susceptibility, while early and standardized propranolol therapy plays a decisive role in reducing treatment duration and recurrence risk. Future studies exploring how hormonal and hypoxia-related pathways interact with microenvironmental factors may provide deeper insights into personalized treatment strategies for IH.

## Data Availability

The datasets presented in this study can be found in online repositories. The names of the repository/repositories and accession number(s) can be found in the article/Supplementary Material.
